# A patient with extensive cerebral calcification due to pseudohypoparathyroidism: a case report

**DOI:** 10.1186/s12902-019-0475-z

**Published:** 2019-12-19

**Authors:** S. W. De Silva, S. D. N. De Silva, C. E. De Silva

**Affiliations:** Sri Jayawardenapura General Hospital, Thalapathpitiya, Nugegoda, Sri Lanka

**Keywords:** Cerebral calcifications, Pseudohypoparathyroidism, Vitamin D deficiency

## Abstract

**Background:**

Pseudohypoparathyroidism(PHP) is a heterogeneous group of disorders due to impaired activation of c AMP dependant pathways following binding of parathyroid hormone (PTH) to its receptor. In PHP end organ resistance to PTH results in hypocalcaemia, hyperphosphataemia and high PTH levels.

**Case presentation:**

A 59 year old male presented with a history of progressive impairment of speech and unsteadiness of gait for 1 week and acute onset altered behavior for 1 day and one episode of generalized seizure. His muscle power was grade four according to MRC (medical research council) scale in all limbs and Chovstek’s and Trousseau’s signs were positive. Urgent non contrast computed tomography scan of the brain revealed extensive bilateral cerebral and cerebellar calcifications. A markedly low ionized calcium level of 0.5 mmol/l, an elevated phosphate level of 9.5 mg/dl (reference range: 2.7–4.5 mg/dl) and an elevated intact PTH of 76.3 pg/l were noted. His renal functions were normal. His hypocalcemia was accentuated by the presence of hypomagnesaemia. His 25 hydroxy vitamin D level was only marginally low which could not account for severe hypocalcaemia. A diagnosis of pseudohypoparathyroidism without phenotypic defects, was made due to hypocalcaemia and increased parathyroid hormone levels with cerebral calcifications. The patient was treated initially with parenteral calcium which was later converted to oral calcium supplements. His coexisting Vitamin D deficiency was corrected with 1αcholecalciferol escalating doses. His hypomagnesaemia was corrected with magnesium sulphate parenteral infusions initially and later with oral preparations. With treatment there was a significant clinical and biochemical response.

**Conclusion:**

Pseudohypoparathyroidism can present for the first time in elderly resulting in extensive cerebral calcifications. Identification and early correction of the deficit will result in both symptomatic and biochemical response.

## Background

Pseudohypoparathyroidism (PHP) is a heterogeneous group of disorders due to impaired activation of c AMP dependant pathways following binding of parathyroid hormone (PTH) to its receptor [1]. In PHP end organ resistance to PTH results in hypocalcaemia, hyperphosphataemia and high PTH levels.

PHP has been classified into PHP-Ia, PHP-Ib, PHP-II and PPHP (pseudopseudohypoparathyroidism). In PHP-I, there is a blunted urinary cyclic AMP response to administration of exogenous PTH. Patients with PHP-I are divided into type a and b. Type a are patients with AHO (Albright hereditary osteodystrophy) and reduced amounts of Gsα in erythrocytes. Type b are patients with absent AHO and with normal amounts of Gsα in erythrocytes. In type II PHP urinary cyclic AMP response to exogenous PTH is normal. In PPHP there are features of AHO but no biochemical abnormalities. AHO is a clinical entity which is characterized by brachydactyly, rounded face, short stature, central obesity, subcutaneous ossifications, and variable degrees of mental retardation [2]. Genetic basis of PHP is loss of function mutation in guanine nucleotide binding protein G-s alpha subunit (GNAS) gene [3, 4].

We present a patient who presented with seizures and various other neurological manifestations due to PHP with extensive cerebral calcification.

## Case presentation

A 59 year old male, presented to Sri Jayawardenapura hospital in February 2017 with a history of progressive impairment of speech, unsteady gait and reduced intake of food over a week. He had had an altered level of behavior for 1 day and a generalized seizure lasting for less than 5 minutes where he was admitted to the local hospital. He denies any limb weakness, difficulty in swallowing, loss of sensation, urinary or bowel incontinence.

There was no history of vomiting, headache, photophobia or fever. He had no past history of chronic kidney disease, gastric or intestinal surgery or chronic diarrhoea. Apart from haematinics which were prescribed to him for iron deficiency anemia due to hemorrhoids, he was not on any regular medication.

His brother was affected with an adult onset seizure disorder and had had cerebral calcification. He was a retired lecturer, with a good family support and reasonable economic background. He was a non smoker and did not consume alcohol.

**Fig. 1:**
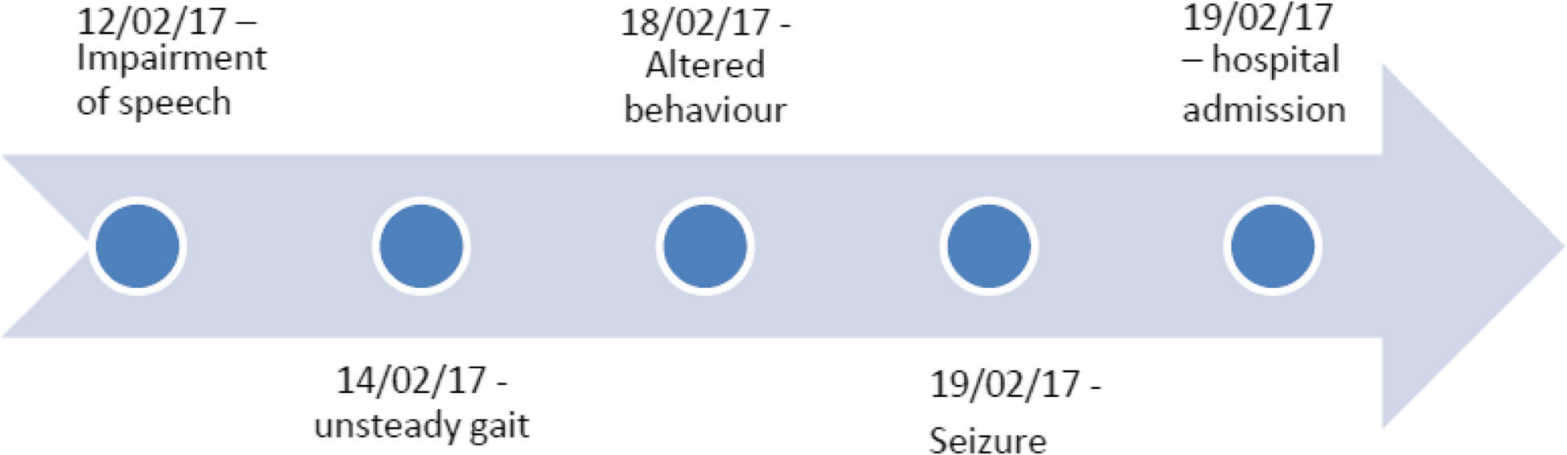
Timeline of history of the patient

On general examination he was obese with a body mass index of 28 kgm^2^. Mild pallor was noted but he was afebrile and there were no bradymetarcapals or metartasals. Neurological examination revealed a Glasgow Coma Scale (GCS) of 14/15. Neck stiffness and Kernig’s sign were absent. Muscle power of grade four was noted in all limbs. Muscle tone was slightly high in all the limbs. Chovstek & Trousseau’s signs were positive. Abdominal, Cardiovascular and respiratory examination was unremarkable. The following figure (Fig. 1) shows the timeline of history of the patient.

The above table (Table 1) shows the summary of investigations of the patient. Diagnostic evaluation revealed severe hypocalcemia, hyperphosphatemia and elevated serum intact PTH level. Serum magnesium was marginally low and 25-hydroxyvitamin D was in insufficient range. His electro cardiogram showed T inversions in lead V1 to V3 and the QT interval was normal. His Chest x- ray was normal and hand x-ray revealed osteopenia but no soft tissue calcifications or bradymetarcarpals.

**Table 1:** Table 1

Investigation (unit)	Value	Reference range
Serum ionized calcium (mmol/l)	0.5	1.12–1.23
Serum phosphate (mg/dl)	9.5	2.7–4.5
Serum magnesium (mg/dl)	1.4	1.7–2.7
Urinary calcium creatinine ratio	0.01	
Serum creatinine (μmol/l)	75	
Creatine phosphokinase/ CPK (U/L)	1294	38–174
Serum intact parathyroid hormone (pg/ml)	76.3	10.4–66.5
25-hydroxyvitamin D (ng/ml)	22.1	30–100
Haemoglobin (g/dl)	8.01	
Alanine transaminase/ALT (U/L)	34	
Aspartate transaminase/ AST (U/L)	97	
Alkaline phophatase/ALP (U/L)	235	
Total Bilirubin (mg/dl)	0.6	
Erythrocyte sedimentation rate/ ESR (mm in 1st hour)	33	
Thyroid stimulating hormone/ TSH (mIU/L)	2.3	0.4–4

**Fig. 2:**
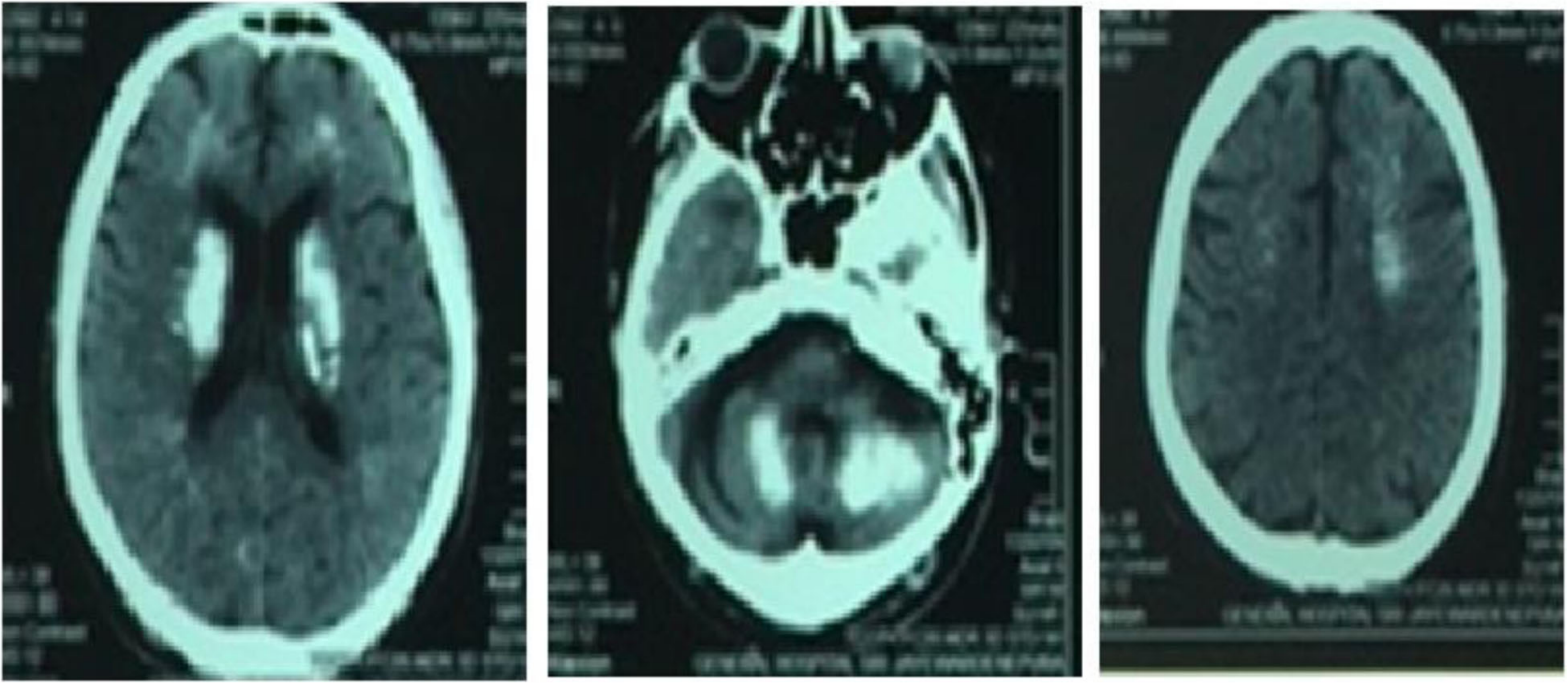
Non contrast CT scan of the brain revealing extensive cerebral calcifications in cerebral cortex and cerebellar hemispheres

**Fig. 3:**
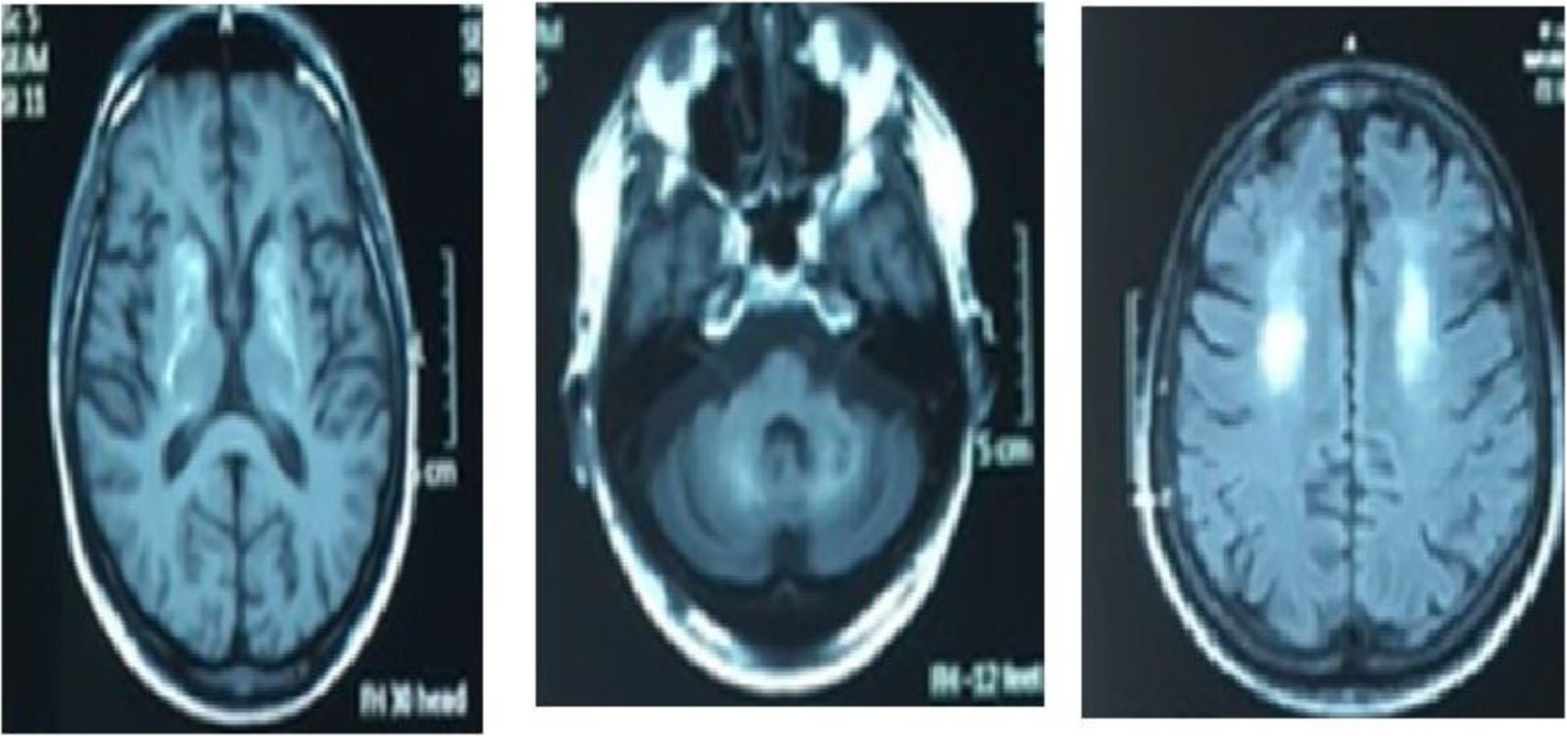
Shows the Magnetic resonance imaging (MRI) scan of the brain revealing bilateral basal ganglia thalamic and dentate ganglia calcification

The below figures represent the imaging findings of our patients. Figure 2 shows the Non contrast CT scan of the brain revealing extensive cerebral calcifications in cerebral cortex and cerebellar hemispheres and Fig. 3 shows the Magnetic resonance imaging (MRI) scan of the brain revealing bilateral basal ganglia thalamic and dentate ganglia calcification.

The patient was managed with a multidisciplinary approach involving internist, neurologist and endocrinologist. Since there was an obvious precipitating factor for the seizure anti epileptic therapy was considered unnecessary and attention was focused on correcting metabolic derangements which precipitated seizures. Patient’s severe symptomatic hypocalcaemia was initially corrected with intravenous 10% calcium gluconate 10 ml over 10 min followed by an infusion of 50 mg elemental calcium per hour for 24 h. Later oral supplements were given as 1200 mg of elemental Calcium in 3 divided doses. He was also replaced with parenteral magnesium sulphate 2 g over 20 min for the coexisting contributory hypomagnesaemia. Patient was commenced on 1αcholecalceferol 0.25 *μ* g three times a day and gradually increased to 1 *μ* g three times a day over few days along with calcium and magnesium supplements.

Patient’s clinical state was monitored 4 hourly and biochemical parameters were monitored daily. Patient did not have further seizures and tetany. Chovsteck’s and Trousseau’s signs and confusion resolved. He did not have any further episodes of altered behavior. Slowness of speech continued for about 2 weeks which later gradually improved. Serum ionized calcium levels gradually improved from 0.5 to 0.93 mmol/l. Inorganic phosphate levels reduced from 9.5 mg/dl to 5.3 mg/dl, intact PTH reduced from 76.3 pg/ml to 67.7 pg/ml. Serum magnesium levels improved from 1.4 mg/dl to 1.9 mg/dl. With the resolution of tetany CPK levels reduced from 1294 U/L to 574 U/L. These changes occurred over 1 week.

Upon both clinical and biochemical response patient was discharged with calcium carbonate 1 g three times a day, elemental magnesium 100 mg three times a day as magnesium carbonate and 1αcholecalceferol 1 μg three times a day. The therapeutic challenge faced during follow up was to maintain a low normal calcium level to prevent nephrocalcinosis.

## Discussion

Differential diagnoses in this patient who presented with symptomatic hypocalcemia were hypoparathyroidism, secondary hyperparathyroidism due to long standing vitamin D deficiency, PTH resistance due to magnesium deficiency and pseudohypoparathyroidism. Elevated serum intact PTH level narrowed down the differentials to the latter three diagnoses. Secondary hyperparathyroidism was considered unlikely in the absence of conditions leading to longstanding vitamin D deficiency such as chronic kidney disease, gastric or intestinal surgery and chronic diarrhoea suggestive of Chron’s disease or celiac disease. Severe hypocalcaemia and high PTH level couldn’t be explained solely by marginally low (insufficient) Vitamin D level. Although severe hypomagnesemia is well known to cause hypocalcaemia through a combination of defective PTH secretion and some degree of PTH resistance mild hypomagnesemia is unlikely to cause isolated PTH resistance leading to such severe hypocalcaemia [5]. Also cerebral calcification can’t be explained by hypomagnesemia alone. The presence of severe hypocalcaemia and elevated PTH with a marginally low vitamin D level and especially the basal, thalamic and dentate ganglia calcification suggests the diagnosis of pseudohypoparathyroidism. Although PHP presenting at 59 years of age is uncommon there have been previous reports of adults presenting with symptomatic hypocalcaemia due to PHP [6].

Elevated alkaline phosphatase is thought to be due to development of osteomalacia secondary to reduced conversion of 25- hydroxyvitamin D to its active form i.e. 1, 25- dihydroxyvitamin D in the absence of PTH action and concurrent vitamin D deficiency [7].

The radiological findings of basal ganglia calcification seen in this case are well described in PHP [8]. However cortical calcification is a rare finding [9] and this is the first such report from Sri Lanka. Other differential diagnoses for this presentation are familial idiopathic basal ganglia calcification (Fahr’s disease [10]), neoplastic, vascular, infectious, and congenital causes, as well as other endocrine/metabolic diseases, including diabetes mellitus, hypoparathyroidism [11], and pseudohypoparathyroidism. However hypocalcaemia, hyperphosphataemia and increased PTH seen in this patient favor the diagnosis of PHP.

Acrodysostosis is another rare genetic disorder which is associated with PTH resistance [12]. However absence of characteristic phenotypic features (underdeveloped facial bones, abnormally small hands and feet) in this patient makes it highly unlikely.

Perera et al. described behavioral changes in patient with pseudohypoparathyroidism [13]. However her non contrast CT brain did not show any cerebral calcification. Therefore metabolic derangements seen in PHP could cause neuropsychiatric manifestations even in the absence of cerebral calcification. Kim et al. described a patient with PHP who presented with focal seizures and was found to have extensive cortical and sub cortical calcifications [9]. Song et al. described a patient who presented with Parkinsonism due to PHP induced basal ganglia calcifications [14]. Unsteady gait seen in our patient may also have been due to basal ganglia calcification.

In order to determine the type of PHP urinary cAMP response to exogenous PTH should have been measured and genetic studies to detect GNAS mutation should have been done. However due to logistic reasons these investigations were not done. However due to the lack of hallmark phenotypic feature of bradymetacarpals this is unlikely to be type Ia. So this patient probably had type Ib or type II PHP. However PHP type II is a rare disorder and only a few cases have been reported to date [15]. Furthermore this patient didn’t have evidence of resistance to other hormones which act via Gs-coupled receptors (normal TSH) which also favours the diagnosis of PHP Ib.

The below table (Table 2) is a comparison of our patient with published cases of PHP. Our case is unique because it highlights the rare occurrence of cerebral cortical calcifications in a patient with PHP without AHO phenotype presenting with speech and gait abnormalities, altered behavior and seizures. This is the first reported such case in Sri Lanka and one of the very few cases reported worldwide.

**Table 2:** Table 2

	Our case report	Ye Sel Kim etal	Perera et al	Song CY etal
Clinical presentation	Progressive slowness of speech, unsteady gait, acute onset altered behavior, generalized seizure	Focal Seizures	Behavioral changes	Clinical manifestations of acute parkinsonism
Radiological Findings	Extensive cerebral cortical,cerebellar, bilateral basalganglia and thalamic calcification	Cortical and sub-cortical calcifications with basalganglia,thalami and cerebellar calcifications.	Normal CT brain	Basal ganglia calcifications
Important biochemistry	Hypocalcemia,hyperphosphatemia,elevated serum intact PTH, marginally low Vitamin D levels	Hypocalcemia,Hyperphosphatemia elevated serum intact PTH	Hypocalcemia,high normal serumphosphate levels,marked elevation of serum intact PTH levels.	Hypocalcemia and hyperphophatemia
Diagnosis	Pseudohypoparathyroidism	Pseudohypoparathyroidism	Pseudohypoparathyroidism	Pseudohypoparathyroidism
Outcome	Responded to calcium and vitamin D supplementation.	Clinical and biochemical response to Calcium and vitamin D Suplimentation	Not available	Marked improvement of dyskinesias with calcium supplimentation
Other Remarks	First reported case of PHP with cortical calcifications in Sri lanka	Rare presentation of PHP with cortical and subcortical calcifications		

### Strenghts

Prompt diagnosis and management of pseudohypoparathroidism lead to an excellent outcome in this patient.

### Limitations

The GNAS mutation and the urinary cAMP response to exogenous PTH were not performed due to logistic and financial reasons.

## Conclusions

Pseudohypoparathyroidism can present for the first time in elderly resulting in extensive cerebral calcifications. Identification and early correction of the deficit will result in both symptomatic and biochemical response.

## Data Availability

Not applicable.

## Abbreviations

AHO: Albright hereditary osteodystrophy
CPK: Creatine phosphokinase
CT: Computed tomography
e-GFR: Estimated glomerular filtration rate
MRI: Magnetic resonance imaging
PHP: Pseudohypoparathyroidism
